# Anti-SARS-CoV-2 IgM/IgG antibodies detection using a patch sensor containing porous microneedles and a paper-based immunoassay

**DOI:** 10.1038/s41598-022-14725-6

**Published:** 2022-07-01

**Authors:** Leilei Bao, Jongho Park, Boyu Qin, Beomjoon Kim

**Affiliations:** Institute of Industrial Science, The Univeristy of Tokyo, 4-6-1 Komaba, Meguro-ku, Tokyo, 153-8505 Japan

**Keywords:** Engineering, Biomedical engineering, Biosensors

## Abstract

Infectious diseases are among the leading causes of mortality worldwide. A new coronavirus named severe acute respiratory syndrome corona virus 2 (SARS-CoV-2) was identified in Wuhan, China in 2019, and the World Health Organization (WHO) declared its outbreak, coronavirus disease 2019 (COVID-19), as a global pandemic in 2020. COVID-19 can spread quickly from person to person. One of the most challenging issues is to identify the infected individuals and prevent potential spread of SARS-CoV-2. Recently, anti-SARS-CoV-2 immunoglobulin M (IgM) and immunoglobulin G (IgG) antibody tests using immunochromatographic methods have been used as a complement to current detection methods and have provided information of the approximate course of COVID-19 infection. However, blood sampling causes pain and poses risks of infection at the needle puncture site. In this study, a novel patch sensor integrating porous microneedles and an immunochromatographic assay (PMNIA) was developed for the rapid detection of anti-SARS-CoV-2 IgM/IgG in dermal interstitial fluid (ISF), which is a rich source of protein biomarkers, such as antibodies. Biodegradable porous microneedles (MNs) made of polylactic acid were fabricated to extract ISF from human skin by capillary effect. The extracted ISF was vertically transported and flowed into the affixed immunoassay biosensor, where specific antibodies could be detected colorimetrically on-site. Anti-SARS-CoV-2 IgM/IgG antibodies were simultaneously detected within 3 min in vitro. Moreover, the limit of detection of anti-SARS-CoV-2 IgM and IgG concentrations was as low as 3 and 7 ng/mL, respectively. The developed device integrating porous MNs and immunochromatographic biosensors is expected to enable minimally invasive, simple, and rapid anti-SARS-CoV-2 IgM/IgG antibody testing. Furthermore, the compact size of the MN and biosensor-integrated device is advantageous for its widespread use. The proposed device has great potential for rapid screening of various infectious diseases in addition to COVID-19 as an effective complementary method with other diagnostic tests.

## Introduction

At the end of 2019, a novel coronavirus, severe acute respiratory syndrome coronavirus 2 (SARS-CoV-2), was identified, which causes coronavirus disease 2019 (COVID-19). It spread worldwide within 3 months owing to its high infectivity^1,2^. In March 2020, the World Health Organization (WHO) announced the COVID-19 outbreak as a global pandemic^3^. A COVID-19 infection spreads quickly from person to person and its symptoms include fatigue, cough, fever, dyspnea, anosmia, and ageusia; more severe symptoms include respiratory insufficiency, which can be life-threatening^4,5^. Furthermore, the rate of asymptomatic infections is reported as 16–38%, which brings difficulties in identifying all the individuals with SARS-CoV-2 infected^6^. COVID-19 vaccines are effective in reducing infection risk and virus transmission; however, the proportion of the population fully vaccinated against COVID-19 remains less than 10% in several low-income countries^7,8^. Therefore, one of the current global challenges is to identify both symptomatic and asymptomatic patients as soon as prevent potential spread of SARS-CoV-2.

Currently, real-time reverse transcription polymerase chain reaction (RT-PCR) is the predominant detection method and remains the gold standard for COVID-19 diagnosis^9^. However, there are certain drawbacks associated with this method that impede rapid COVID-19 detection: (1) substantial laboratory infrastructure and well-equipped facilities are necessary to conduct real-time RT-PCR; (2) well-trained medical technicians are required for collecting naso- or oropharyngeal swab samples and operating sophisticated laboratory instruments; (3) the total turnaround time for real-time RT-PCR testing is long (4–6 h), which poses a risk of cross-contamination^10^. Thus, being expensive and time-consuming, and requiring medical personnel, using real-time RT-PCR diagnosis of COVID-19 is not feasible in many resource-limited countries.

In addition to detecting SARS-CoV-2 in samples collected from the back of the nose and throat, testing of anti-SARS-CoV-2 immunoglobulin M (IgM) and immunoglobulin G (IgG) antibodies is a good alternative for confirmation of COVID-19 infection^11–13^. According to the previous research, IgM and IgG generated by SARS-CoV-2 could be detected in 31.8–40.9% of patients at 0–5 days after symptom onset^14^, while 94% and 100% of patients were tested positive for anti-SARS-CoV-2 IgM and IgG, respectively, within 3 weeks after symptom onset^15^. Moreover, the stage of COVID-19 infection can be approximately known, because anti-SARS-CoV-2 IgM antibody is the first antibody response to initial exposure to SARS-CoV-2 antigens, which increases within a week after symptom onset, peaks after 2–3 weeks, and then decreases to a low level in most patients^16^. On the contrary, a plateau in IgG level was observed at 3 weeks after symptom onset, and the level rose to peak between 3 and 8 weeks and maintained at a high level for a long time^16^. Therefore, detection of both IgM and IgG can provide conclusive information about the approximate course of COVID-19 infection. Additionally, according to the previous study about anti-SARS-CoV-2 spike protein receptor binding domain (RBD) IgG in blood samples, its concentration ranged 331–25.7 µg/mL after onset of symptoms^17^, while the concentration of the target IgG in convalescent serum was 7–2100 ng/mL^18^. Besides, IgM against SARS-CoV-2 spike RBD in plasma/serum indicated similar concentration with IgG after onset of symptoms^19^. Nowadays, lateral-flow immunochromatographic assay (LFIA) strips based on gold nanoparticles (AuNPs) are being widely used for rapid detection of SARS-CoV-2-specific antibodies^20–26^. Usually, when using the self-testing COVID-19 LFIA, individuals have to collect their blood sample using a lancing device and then immediately pipet it into the cassette sample well^27^. Then, 2–3 drops of running buffer are added to the sample well for diluting the specimen. The diluted blood sample flows through the test strip, and the colorimetric label can be read within 10–20 min. Owing to simple operation, and not requiring complex instruments or steps, LFIA strips for testing anti-SARS-CoV-2 antibodies have emerged as a reliable platform for point-of-care (POC) testing in many countries. More importantly, SARS-CoV-2-specific antibodies could be detected in asymptomatic patients as well as individuals with negative RT-PCR results who had close contacts with COVID-19-infected patients^15^, and thus, target antibody detection can be used as a complement to real-time RT-PCR testing. However, bleeding caused by finger prick may cause pain and increase the risk of infection or even cross-contamination^28^. Moreover, patients have to dispose of the test kit components, which are a potential biohazardous waste. Thus, minimally-invasive and easy-to-use methods are urgently needed to achieve rapid detection of SARS-CoV-2-specific antibodies.

Over the past few decades, there has been a growing trend toward using microneedles (MNs). In 1998, MNs were initially developed to demonstrate the feasibility of painless transdermal drug delivery^29^. The micro-sized structure of MNs enables them to penetrate the human skin without stimulating nerve endings, which makes the process painless with minimal and better patient compliance^30^. Recently, MNs have emerged as a powerful platform for interstitial fluid (ISF) sampling as well as for biosensing and monitoring of various types of biomarkers, such as glucose^31–34^, cholesterol^35,36^ and protein biomarkers^37–39^. Till now, hollow, swellable, and porous MNs have been developed for ISF extraction. However, hollow MNs with an internal conduit are commonly made of silicon^40^, metal^36^, or non-dissolving polymer^38,41^; hence, if broken in the skin, they may harm the human body. Swellable MNs usually require post-processing to recover the target analyte via centrifugation or solvent extraction^34,35^. Here, porous MNs could resolve those problems. Recently, porous MNs with interconnected microspores inside the entire MN structure have been focused. Porous MNs have been developed within a decade for collecting the dermal ISF whereas various biocompatible and biodegradable polymers have been successfully used to fabricate of porous structures^42^. The ISF extraction can be realized by capillary action owing to micron-sized porous structures and moreover, lab-on-chips biosensing device can be directly affixed to the porous MNs for subsequent analysis and disease diagnosis^31,43^. Therefore, an immunoassay biosensor can be designed and integrated with porous MNs for simple and rapid detection of IgM and IgG antibodies against SARS-CoV-2 in the ISF. However, because of the continuous voids inside the MN, the MN structure is more fragile and thus there is a trade-off between the porosity and the mechanical strength.

By far, blood sampling has been predominant in ex vivo analysis. Other peripheral biofluids such as tears, sweat, saliva and urine have been used for alternative sample sources but the level of biomarkers indicated the poor correlation with that of blood^34,44^. However, other body fluid like ISF which is located in the epidermis and dermis layers of human skin has not been extensively embraced in medical applications due to the difficulties in minimally-invasive sampling and sufficient collection for the subsequent ex vivo analysis^39,44^. Meanwhile, it is reported that ISF, primarily located in the epidermis and dermis layers of human skin, contains a wide range of metabolites and proteins (e.g., antibodies) that demonstrates close correlation with those in blood^13,37^. In addition, it is reported that the antibody level in ISF is about 15–25% of that in blood^45^. Therefore, the concentration of anti-SARS-CoV-2 IgM/IgG antibody in ISF is supposed to be in the range of 1–6.4 µg/mL, and it is feasible to detect target IgM and IgG in ISF to substitute for blood sampling.

In this study, we developed a novel patch sensor (size: 1.5 cm × 3.5 cm) integrating biodegradable porous MNs and an immunochromatographic assay (PMNIA) for rapid, painless, easy to use, and simultaneous detection of anti-SARS-CoV-2 IgM and IgG antibodies in ISF. The innovation of our newly developed device is embodied in two aspects. First, we achieved antibody detection without blood sampling and propose a new approach to fabricate biodegradable porous MNs with emulsion droplets. Polylactic acid (PLA) microspheres prepared from a single emulsion were used to form continuous micropores, and subjected to heat treatment to bond them together. Moreover, we found that the porous MNs showed optimal sample fluid extraction by capillary effect and effective skin penetration after 30 min heating at 180 °C. Second, a newly designed paper-based colloidal gold immunoassay biosensor was developed. When transported from the porous MNs to the biosensing platform, the sampled ISF could flow vertically and autonomously from the sample pad (bottom layer), conjugate pad, nitrocellulose (NC) membrane (top layer), and finally to the absorption pad by capillary action, and antibody testing was completed within 3 min by visual observation of the colored bands. Moreover, the limit of detection (LoD) of the proposed device was 3 ng/mL for IgM and 7 ng/mL for IgG, which shows the advantages of fast screening of COVID-19 over the current commercial LFIAs.

## Results

### Design of the PMNIA for COVID-19 detection

**Figure 1:**
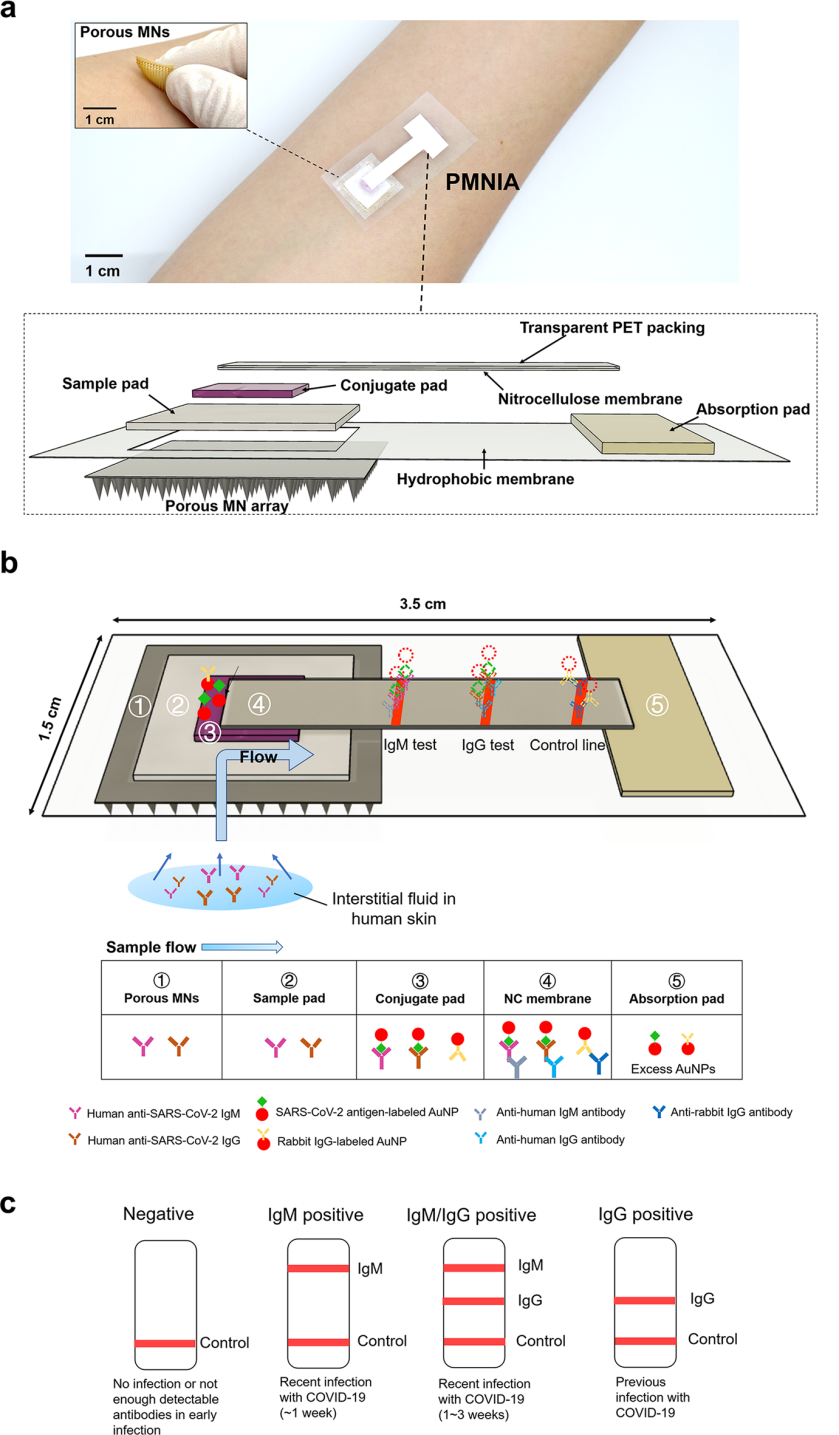
Design of PMNIA for COVID-19 detection. (**a**) Photograph of a structural overview of the detection device and a magnified view of the device comprising the porous MN array and components of the paper-based immunochromatographic biosensor. Inset image shows the porous MN array with a flexible substrate. (**b**) Schematic diagram of the PMNIA principle for simultaneous detection of anti-SARS-CoV-2 IgM/IgG. (**c**) Illustration of the interpretation of the different detection results using PMNIA.

The diagnostic device consisted of a biodegradable porous MN array and novel colloidal gold immunochromatographic biosensor. A structural overview of the assembled device is shown in Fig. 1a and it can be attached to the anterior forearm, which lacks body hair and facilitates MN penetration^46^. Furthermore, the flexibility of the fabricated porous MN patch allows it to fit well with the curvature of the human skin. Briefly, the ISF was extracted by porous MNs and vertically transported to the immunochromatographic biosensor in the order of sample pad, conjugate pad, and one end of NC membrane, and then laterally flowed through the entire NC membrane strip, where the presence or absence of anti-SARS-CoV-2 IgM/IgG was detected and observed colorimetrically.

The porous MN array was made from PLA microspheres prepared by a single emulsion and subsequently subjected to heat treatment to form interconnected micropores to absorb and transport ISF by capillary effect. PLA is a polymer with good biocompatibility and biodegradability that does not harm the human body, and is extensively employed in biomedical applications^47^. The size of the porous MN array was 1.5 cm × 1.5 cm with MNs aligned on an array of 13 × 13 to absorb the ISF sample for biosensing. The porous MN array was then affixed to a hydrophobic membrane (thickness: 38 µm) with a hole (1 cm × 1 cm) to transport the sampled ISF from the MN to the paper-based immunochromatographic assay via capillary action, as well as to prevent the biosensor from being drenched by excess ISF.

The newly designed immunochromatographic biosensor comprises a sample pad, conjugate release pad, NC membrane with a transparent polyethylene terephthalate (PET) packing sheet attached to the back side, and absorption pad. The sample pad was fitted to a punched hydrophobic membrane and interfaced with the substrate of the porous MN array for subsequent sample collection. Here, the SARS-CoV-2 spike protein RBD, which is the main immunogen to induce antibody production against COVID-19^48^, has high specificity for binding anti-SARS-CoV-2 IgM/IgG antibodies^49^. The spike protein RBD was conjugated to colloidal AuNPs, dispensed onto the pretreated conjugate pad, and finally attached to the top of the sample pad. Next, the NC membrane strip was placed on the conjugate pad. Anti-human IgM and IgG antibodies (mouse monoclonal) were immobilized on the IgM and IgG test lines of the NC membrane, respectively, to capture the immunocomplexes in which SARS-CoV-2 spike RBD-conjugated AuNPs bound with anti-SARS-CoV-2 IgM/IgG antibody. Rabbit IgG-conjugated AuNPs were prepared and coated on the same conjugate pad to be captured by an anti-rabbit IgG antibody, which was immobilized on the NC membrane as a control line. Absorption pad was affixed to the distal end of NC membrane strip to absorb excess ISF and maintain the continuous sample flow. The final size of the diagnostic device after assembly was 1.5 cm × 3.5 cm and the material and size of each functionalized “paper” is shown in Table 1.

**Table 1:** Properties and design of each “paper” component used in the immunochromatographic biosensor.

	Sample pad	Conjugate pad	Nitrocellulose (NC) membrane	Absorption pad
Material	Glass fiber	Glass fiber	NC membrane with transparent PET packing sheet	100% cotton linter
Size	1 cm × 1 cm	5 mm × 5 mm	4 mm × 2 cm	5 mm × 1–1.5 cm
Function	Absorption and collection of ISF flowing from MN array	1. Preservation of dried antigen-conjugated AuNPs	1. Immobilization of the capture bio-receptor	1. Absorption of excess fluids
		2. Release of labeled AuNPs when wetted by flowed ISF	2. Formation of colorimetric immunecomplexes for visual observation	2. Guarantee of continuous and complete fluid migration

The principle of the COVID-19 diagnostic device is shown in Fig. 1b. After penetrating the human skin, the porous MN array extracts and transports ISF through continuous micropores to the substrate via capillary action. Subsequently, the extracted ISF is absorbed by the sample pad and moved vertically to the conjugate pad located above the sample pad. If anti-SARS-CoV-2 IgM and IgG antibodies are present in the sampled ISF, they would bind SARS-CoV-2 spike protein RBD-labeled AuNPs dispensed on the conjugate pad. The AuNP-antibody conjugates migrate vertically to the NC membrane. Because the NC membrane faces the conjugate pad, the conjugates continue to flow laterally through the entire strip. Subsequently, anti-SARS-CoV-2 IgM antibodies are captured by anti-human IgM antibodies immobilized on the IgM line, while anti-SARS-CoV-2 IgG antibodies are captured by anti-human IgG antibodies immobilized on the IgG line. The presence of anti-SARS-CoV-2 IgM and IgG antibodies is indicated by colored lines, which can be read through the transparent PET packing sheet. If the sampled ISF does not contain specific antibodies against SARS-CoV-2, no immunocomplexes are formed and therefore, no colorimetric labels are be observed. Excess AuNPs conjugated with rabbit IgG are captured by the anti-rabbit IgG antibody immobilized on the control line. The appearance of a colored control line indicates that the sampled ISF migrated through the immunoassay, and the device operated optimally. Finally, the remaining fluid is collected by the absorption pad by capillary force, which provides a sufficient bed volume for the complete flow of the sampled ISF. As illustrated in Fig. 1c, the detection results can be read and critical information be obtained about the course of the COVID-19 infection.

### Fabrication of porous PLA MNs

**Figure 2:**
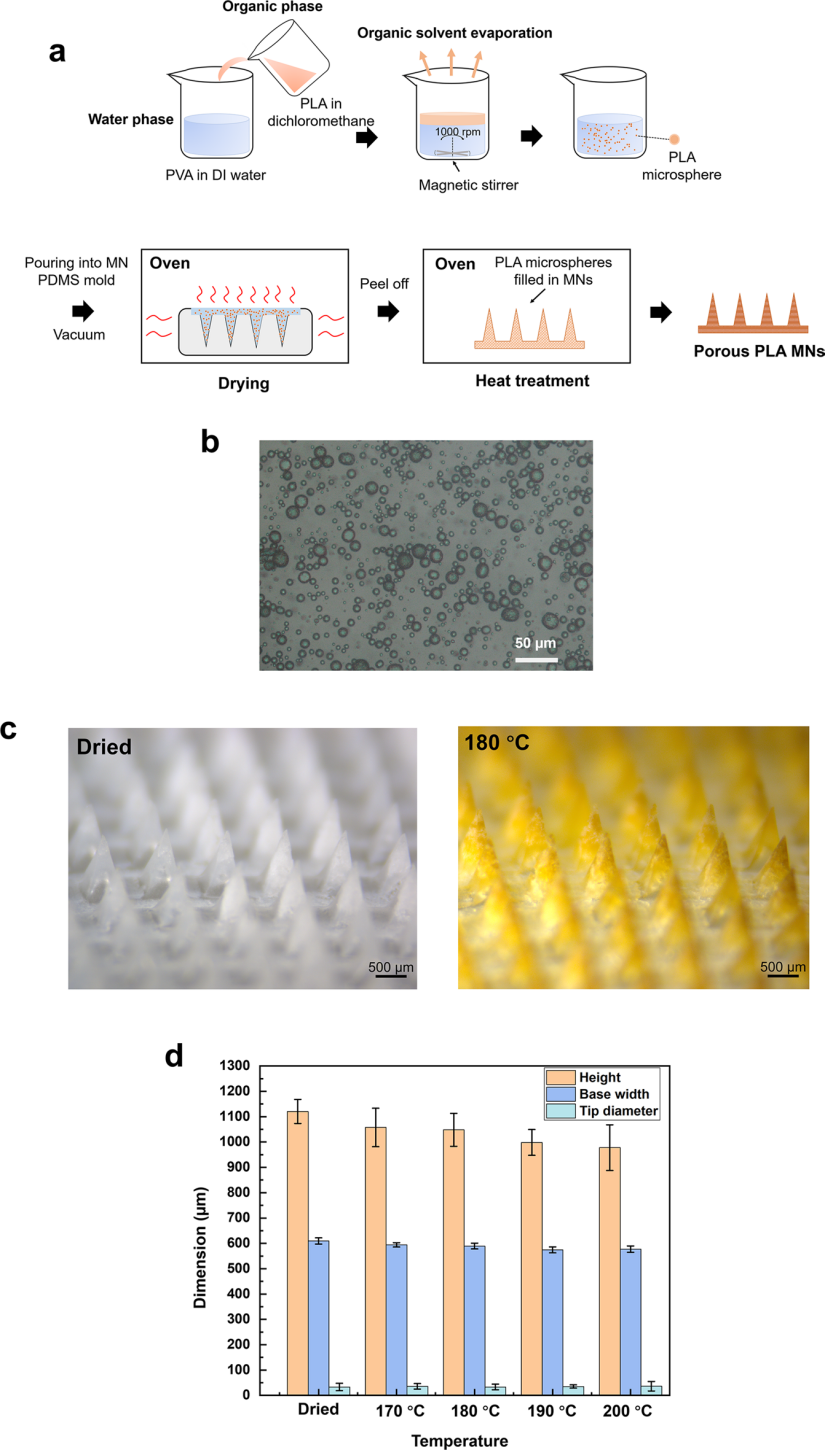
Fabrication of porous PLA MNs with emulsion droplets. (**a**) Single emulsion was used for fabricating PLA microspheres followed by heat treatment for melting and bonding microspheres to form interconnected micropores. (**b**) Fabricated PLA microspheres imaged by optical microscopy. (**c**) Porous PLA MN array before (left) and after treatment (right) at 180 °C for 30 min. (**d**) Dimensions of porous PLA MNs after heat treatment (n = 5).

Several studies have explored the fabrication methods of preparing porous MNs from porogen leaching^31–33,50^. However, the leaching process takes a relatively long time and the materials used for MN fabrication are limited because sufficient mechanical strength should be guaranteed, particularly after the removal of porogens. In this study, a new approach for directly fabricating porous microstructures inside MNs with emulsion droplets has been proposed. Here, PLA, a biodegradable polymer produced from renewable resources^51^, was used to prepare microspheres. PLA microspheres prepared by a single emulsion were used to directly form interconnected micropores inside the MNs (Fig. 2a), followed by heat treatment to bond them together to stabilize the porous structures. The diameter of the fabricated microspheres was 15.5 ± 6.9 μm as determined by optical microscopy (Fig. 2b). Subsequently, the prepared solution was casted into the MN female mold and porous MNs were acquired after dried and peeled off. After heat treatment at four different temperatures (170 °C, 180 °C, 190 °C and 200 °C), the porous PLA MN shapes and dimensions were measured. As indicated in Fig. 2c, the shape of MNs was maintained after heat treatment (MNs after heat treatment at 170 °C, 190 °C and 200 °C for 30 min are shown in Fig. S1), while a difference in the color of the porous MNs was observed before and after heat treatment. We confirmed that the color change was attributed to PVA that was used as a surfactant by comparing fabricated PLA MNs with MNs made from 5% (w/v) PVA only (Fig. S2a). During heat treatment, the polyene fractions formed in the macromolecular PVA chains resulted in a gradual shift of the wavelength toward a longer wavelength^52^. Therefore, compared with the MNs before heat treatment, the whole MN exhibited yellow–brown coloration after heat treatment. In addition, it was supposed that PVA plays two roles in the formation of porous PLA MNs from the results using different PVA solutions (Fig. S2b). One is a surfactant in forming PLA microspheres during single emulsion process. The other is maintaining the shape of MNs during the molding process. Regarding the dimension of fabricated MNs, both the height and width of the MN base decreased slightly after the heat treatment (Fig. 2d). This shrinkage could be attributed to the melting and bonding of the PLA microspheres inside the MN structures.

### Porous structures and fluid absorbing capability

**Figure 3:**
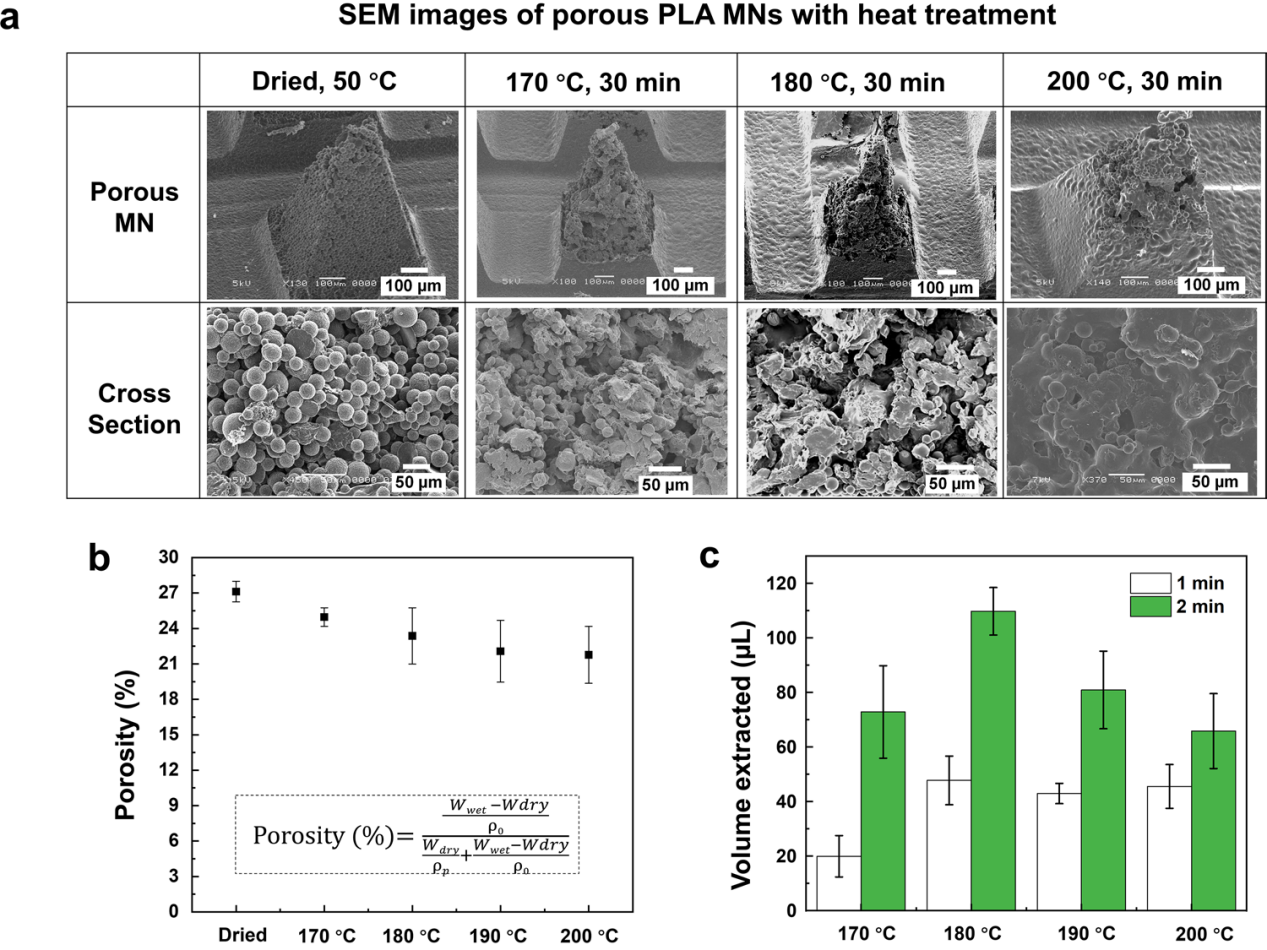
Porous structure and fluid absorption of MNs after heat treatment. (**a**) SEM images of single MN structure and its cross-section. (**b**) Porosity of porous MNs as determined by water imbibition method. (**c**) The absorbed volume of sample fluids from 1% (w/v) agarose gel at 1 min and 2 min (n = 4).

The influence of the temperature used for heat treatment on the formation of porous structures inside the MNs was investigated using scanning electron microscopy (SEM). The structural and cross-sectional images of a single MN are shown in Fig. 3a. After the PLA microspheres were filled into the cavity of the MN mold and dried at 50 °C for 2 h, continuous voids were formed among the PLA microspheres that were not bonded together. After heat treatment at 170 °C (melting point of PLA) for 30 min, a part of microspheres began to melt and bond together. When the temperature was set at 180 °C, most of the microspheres bonded together, which resulted in the formation of micron-sized interconnected pores. In contrast, microspheres were over-melted, and only a few interconnected voids were confirmed when PLA MNs were heated at 200 °C. These results showed that the heat treatment temperature significantly influenced melting and bonding of PLA microspheres as well as the formation of porous structures.

Next, the porosity of porous PLA MNs was measured using the water imbibition method (Fig. 3b). Before heat treatment, the porosity was 27.2 ± 0.9%. As the temperature for heat treatment increased, the porosity gradually decreased because more PLA microspheres melted and bonded, which contributed to the decrease in continuous voids inside the MNs. Then, the absorbing ability of the fabricated porous PLA MNs after heat treatment was investigated. Agarose gel (1%, w/v) in phosphate-buffered saline (PBS) covered with aluminum foil was used for mimicking human skin^31^. A force of 5 N was applied to the MN array to penetrate the skin model. After heat treatment at 170–200 °C, MNs successfully pierced the aluminum foil and absorbed the sample fluid from the agarose gel. However, the MNs dried at 50 °C penetrated the aluminum foil but failed to maintain the MN structure, and most of the PLA microspheres from MNs collapsed and remained inside the agarose gel. This was attributed to the lack of adhesion between the microspheres inside the MNs. The absorption volumes of the sample fluids for 1 min and 2 min are shown in Fig. 3c. MNs heated at 180 °C had the highest absorption volume of sample fluid within 2 min, which extracted 109.8 ± 8.7 μL sample fluid from the human skin model. Since human skin has a lower water content between 58 and 72%^53^ in comparison with 1% agarose gel containing 99% buffer solution, the porous MNs heat-treated at 180 °C can theoretically extract 64.4–79.8 μL from the human skin within 2 min. Assuming that the extracted fluid cannot infiltrate into the NC membrane and flow through the strip via capillary action until the sample pad and conjugate pad become saturated with the extracted fluid, theoretically at least 63.7 μL ISF is the necessary absorption amount from the human skin (calculated from the total size of sample pad and conjugate pad and intake rate of the used glass fiber pad which is 50.9 μL/cm^2^). Therefore, the porous PLA MN array subjected to 30 min heat treatment at 180 °C could extract sufficient ISF for subsequent immunoassay biosensing.

### Mechanical property and skin penetration

**Figure 4:**
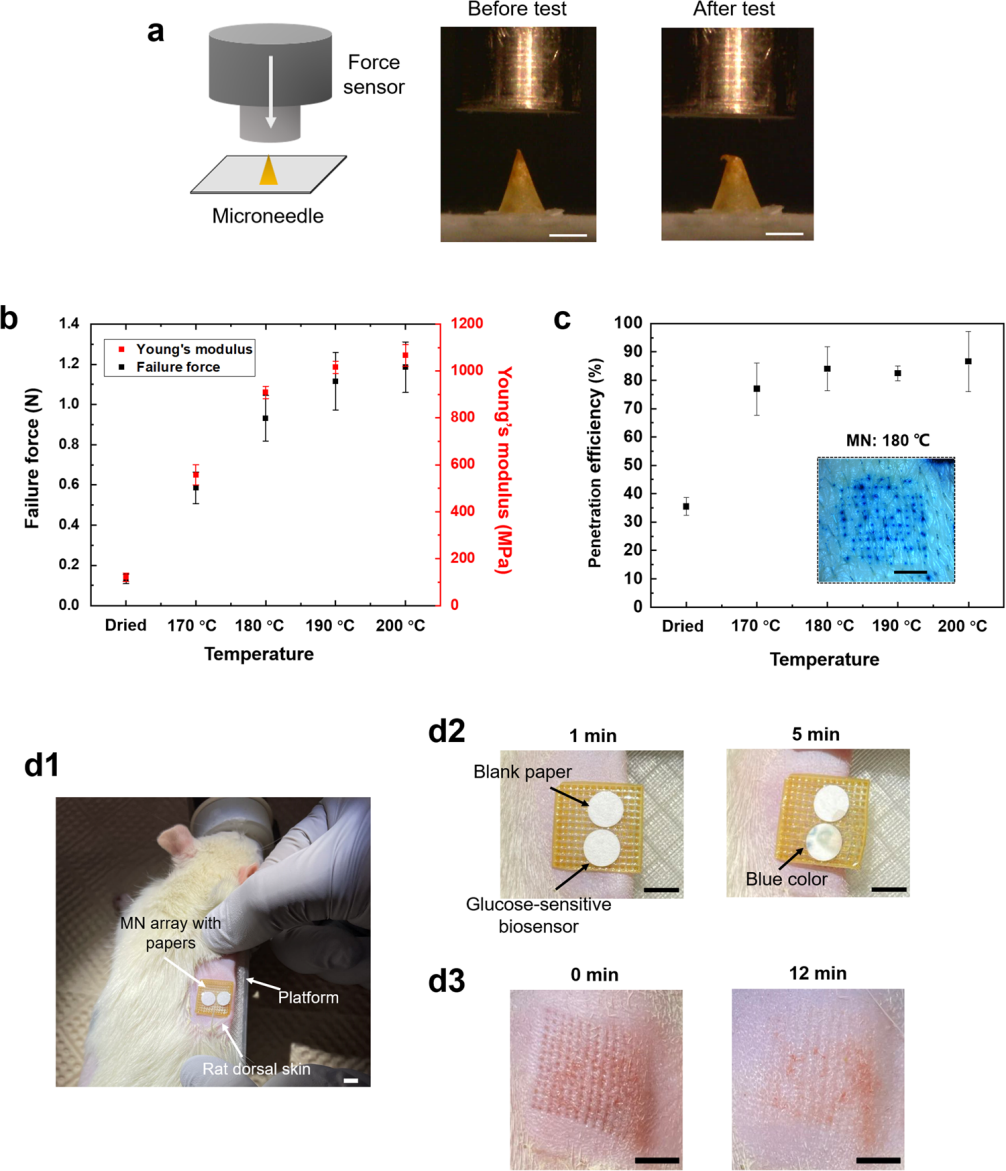
Evaluation of mechanical property and skin insertion of porous MNs. (**a**) A schematic of a mechanical compression test setup with axial moving force sensor and optical micrographs of porous MNs heat-treated at 180 °C before and after an axial failure test. Scale bar, 500 μm. (**b**) Failure force and calculated Young’s modulus of porous MNs after heat treatment at different temperatures. (**c**) Penetration efficiency of porous PLA MNs after heat treatment at different temperatures. Inset image shows the insertion of porous MNs after heat treatment at 180 °C into the porcine skin stained with methylene blue. Scale bar, 5 mm. (**d**) Rat skin insertion and ISF extraction using porous MNs attached with a glucose-sensitive paper and a blank paper. Rat dorsal skin was fixed onto a 3D-printed platform for MN insertion (**d1**) and images indicated ISF extraction by porous MNs and move to the attached papers within 5 min (**d2**) as well as recovery of the rat skin over time after removal of the MN array (**d3**). Scale bar, 5 mm.

The mechanical strength of the porous PLA MNs after heat treatment was measured and analyzed by performing axial compression tests using a force–displacement test station (Fig. 4a). As demonstrated in Fig. 4b, prior to heat treatment, the average failure force of dried porous PLA MN was 0.13 ± 0.02 N, whereas the average failure forces of porous PLA MN after heat treatment at 170 °C, 180 °C, 190 °C, and 200 °C for 30 min were 0.59 ± 0.08 N, 0.93 ± 0. 11 N, 1.12 ± 0.14 N, and 1.19 ± 0.13 N, respectively. The representative force–displacement curves of the porous MNs before and after heat treatment are shown in Fig. S3. The results indicate that porous PLA MNs possessed sufficient mechanical strength (> 0.058 N) for skin puncture, regardless of the temperature at which heat treatment was performed^54^. Moreover, the Young’s modulus (E) of porous PLA MNs could be calculated based on the MN dimensions and results of the failure forces (Eq. (2)). The Young’s modulus of the porous MNs increased markedly from 120.7 ± 18.2 MPa before heat treatment to 908.7 ± 26.4 MPa after heat treatment at 180 °C for 30 min, and the mechanical strength of porous MNs increased at higher heating temperature owing to more PLA microspheres melting and bonding inside the MN structures (Fig. 4b).

Next, an insertion test using porcine skin was conducted to validate whether porous PLA MNs were sufficiently rigid to puncture the skin. The results showed that porous MNs successfully punctured the porcine skin, irrespective of the heat treatment (Fig. S4). However, while the MN patch without heat treatment peeled off from the porcine skin after insertion, the entire structure of the MN patch was not maintained and finally separated (Fig. S4a). This was because the PLA microspheres in the entire MN patch did not adhere to each other, leading to the patch collapse during manual application and eventual separation when it was peeled off from porcine skin. In contrast, most of the MNs subjected to heat treatment above melting temperature successfully punctured the skin (Fig. S4b–d). The results of the penetration efficiency (PE) of porous PLA MNs after heat treatment are shown in Fig. 4c. The PE of porous MNs increased significantly after heat treatment compared with that of porous MNs before heat treatment. Moreover, after heat treatment at 180–200 °C, the MNs showed comparable PEs, indicating that sufficient adhesion of melted PLA microspheres heated at temperatures over 180 °C imparts MNs adequate mechanical strength to puncture the skin and maintain their structure after penetration.

Following in vitro results, we conducted an in vivo test wherein a porous MN array heat-treated at 180 °C was applied to the dorsal skin of a live rat by thumb force. To verify whether the fabricated MNs have capability of extracting, a glucose-sensitive paper biosensor^31^ and a blank paper were attached to the porous MN array with transparent adhesive tape covered as demonstrated in Fig. 4d1 and Fig. S5. After MN insertion for 5 min, the body fluid was extracted by MNs and infiltrated into the blank paper as well as the glucose-sensitive paper biosensor (Fig. 4d2). Besides, colorimetric reaction and blue color development of the biosensor were observed by naked-eyes because of the oxidation of 3,3′,5,5′-Tetramethylbenzidine (TMB) employed as a chromogenic dye (Fig. S5c), indicating the body fluid containing glucose was extracted and transported via MNs to the attached papers by capillary effect of MN porous structures and papers. After removal of the MNs, skin recovery after MN treatment was also examined. As shown in Fig. 4d3, microholes corresponding to MN insertion spots were marked on the rat skin surface and gradually contracted, suggesting rapid skin recovery as well as reduced skin damage after MN application. Subsequently, the prepared luminol solution was sprayed to the blank paper in the dark to confirm whether blood was present in the extracted fluid (Fig. S5d2). As a result, no chemiluminescence reaction was visualized (Fig. S6), suggesting the fabricated MNs could insert into the rat skin and extract the dermal ISF to the sensor layer within 5 min.

### Assembly of the novel patch and sample collection performance

**Figure 5:**
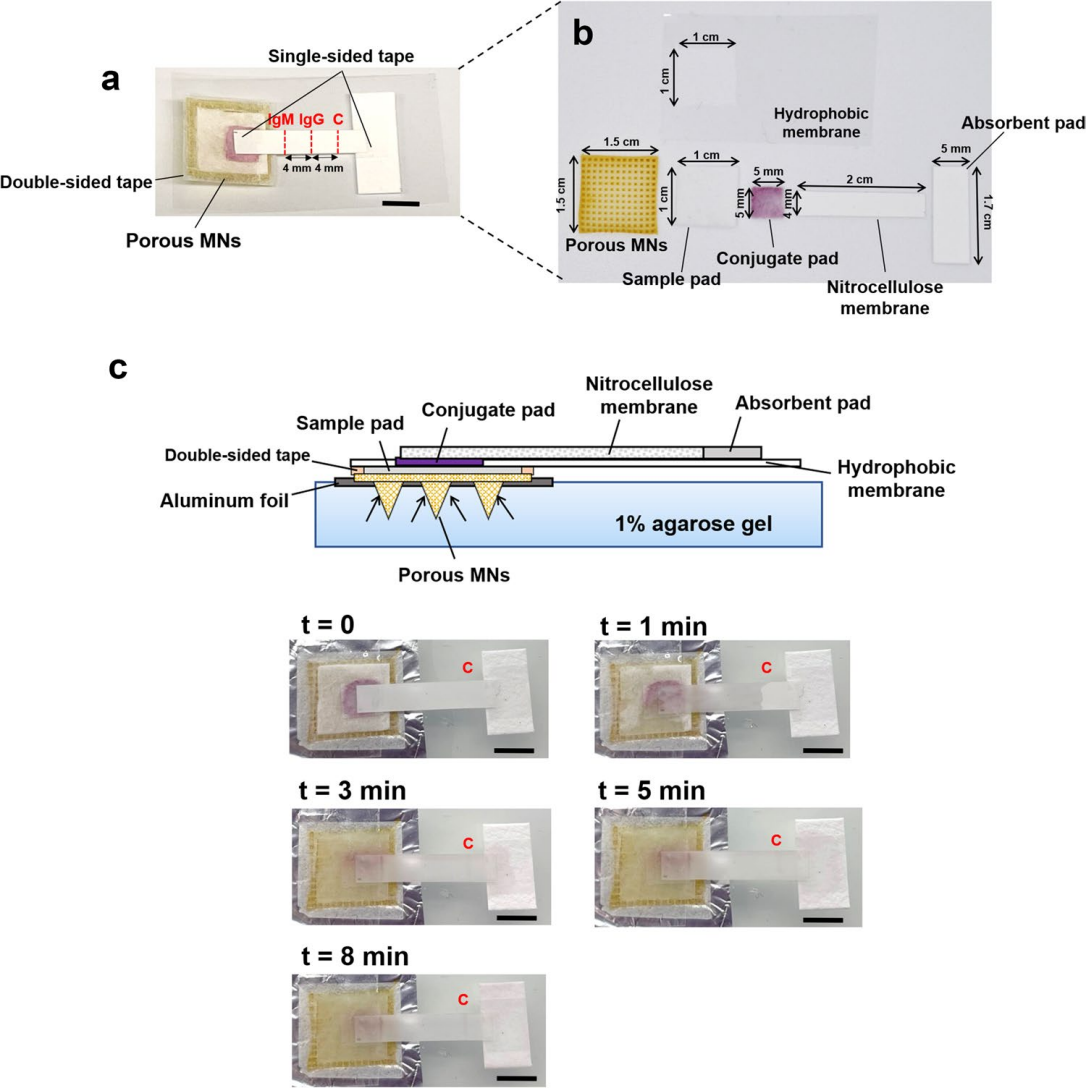
Assembly of the PMNIA as the anti-SARS-CoV-2 antibody detection patch, its fluid extraction capability, and complete flow testing time. (**a**) Image of the assembled detection patch integrated with porous MNs and paper-based immunoassay biosensor. Scale bar, 5 mm. (**b**) Components of the PMNIA assembly. (**c**) Illustration of the PMNIA applied on the skin model and sequential still frame images demonstrating sample fluid extraction by porous MNs and transport through the entire immunoassay biosensor leading to the development of reddish-purple color on the control line. Application time is indicated in the upper left corners. “c” in red represents control line. Scale bar, 5 mm.

As shown in Fig. 5a,b, the porous MN array, the prepared sample pad, conjugate pad, NC membrane, absorption pad and punched hydrophobic membrane were assembled and the PMNIA was formed. The porous MN array was attached to one side of the hydrophobic membrane using a double-sided tape, and the prepared immunoassay was affixed to the other side. Then, transparent single-sided adhesive tape was applied to both ends of the NC membrane to fix the immunoassay structure.

Next, the time required for the complete flow of the detection patch was tested using the human skin model. The MN array was manually applied to the skin model, and the sample fluid was extracted and transported by porous MNs. Soon after, the conjugated pad was wetted by the fluid, which was transported vertically upward from the sample pad by capillary effect, and labeled AuNPs were released from the conjugate pad to the NC membrane (Fig. 5c). The results indicated that within 1 min, the sample fluid was quickly extracted by the MN array and subsequently flowed to the sample pad. After 3 min, the sample fluid flowed through the NC membrane strip and reached the absorption pad. Moreover, the reddish-purple color of the control line indicated the point where the rabbit IgG-conjugated AuNPs passed through the control line zone and were bound to anti-rabbit IgG. Thus, the total time required for the fluid extraction and its complete flow through the entire detection patch with visual confirmation of result was 3 min. In addition, the absorption pad was drenched for 8 min with the control line visualized clearly.

### Anti-SARS-CoV-2 IgM/IgG antibody detection and sensitivity

**Figure 6:**
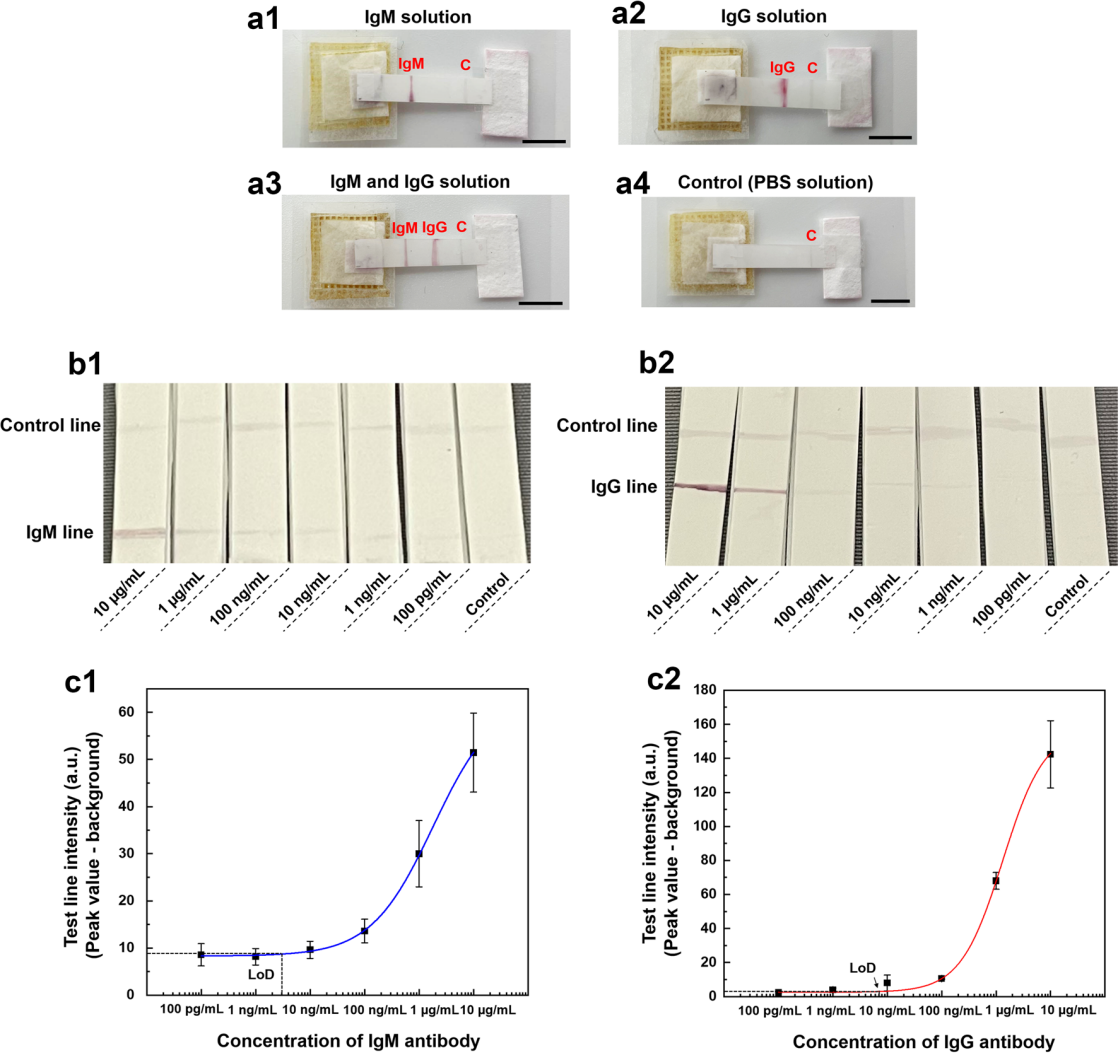
Detection of SARS-CoV-2-specific IgM/IgG antibodies using patch integrated porous MNs and paper-based immunoassay and its sensitivity. (**a**) Visual test results of sample ISF containing IgM (**a1**), IgG (**a2**), mixture of IgM and IgG (**a3**), and blank control (**a4**). Scale bar, 5 mm. (**b**) Detection of anti-SARS-CoV-2 IgM (**b1**) and IgG (**b2**) with different concentrations. (**c**) The average color intensity of IgM line (**c1**) and IgG line (**c2**), and their fitted curves. The graphs were obtained by analyzing three independent detection patches (n = 3) for each target concentration and the fitted curves correspond to the following equation: y = start + (end – start) × x^n^/(k^n^ + x^n^). For IgM detection (**c1**): start = 8.29 ± 0.29, end = 63.24 ± 3.12, k = 1801.30 ± 732.86, n = 0.76 ± 0.09 with R^2^ = 0.998; For IgG detection (**c2)**: start = 2.54 ± 0.43, end = 157.29 ± 33.49, k = 1322.64 ± 534.49, n = 1.12 ± 0.11 with R^2^ = 0.993.

The performance of the anti-SARS-CoV-2 IgM/IgG detection patch was evaluated by conducting four individual measurements. ISF simulant (PBS buffer) containing 0.5 µg/mL anti-SARS-CoV-2 IgM antibody, 0.5 µg/mL anti-SARS-CoV-2 IgG antibody, mixture of 0.5 µg/mL anti-SARS-CoV-2 IgM and IgG antibody solution, and PBS solution as control were prepared and 80 µL of each solution was pipetted onto the porous MN array of the detection devices. As shown in Fig. 6a1–a3, the results indicated that if SARS-CoV-2-specific antibodies were present in the specimen, a red/purple color would appear on the test line. In contrast, if SARS-CoV-2-specific antibodies were not present in the specimen, no immunocomplex was bound at the test lines; however, the control line was colored red/purple, indicating that the fluid flowed adequately along the device, as shown in Fig. 6a4. Moreover, when the specimen contained only anti-SARS-CoV-2 IgM or IgG antibody, the IgM or IgG lines appeared red/purple, respectively, whereas both IgM and IgG lines appeared red/purple when the mixture of anti-SARS-CoV-2 IgM and IgG antibody solution was applied. All four individual measurements were performed three times. In addition, all testing results could be observed within 3 min, suggesting rapid detection of anti-SARS-CoV-2 IgM/IgG antibody using this novel patch.

Next, the sensitivity of the novel immunoassay was investigated using serial dilutions of anti-SARS-CoV-2 IgM and IgG antibody samples for detection. The sample solutions containing IgM and IgG antibodies were prepared in the range of 0.1–10 µg/mL and then 80 µL of each sample solution was used for analysis. Figure 6b1 displays the image of the COVID-19 detection device testing different concentrations of anti-SARS-CoV-2 IgM antibody (0.1–10 µg/mL). The results demonstrated that as the IgM concentration decreased, the reddish-purple color on the IgM line weakened as fewer SARS-CoV-2 spike RBD-AuNPs-IgM immunocomplexes were formed and caught on the IgM lines. The testing results of different concentrations of anti-SARS-CoV-2 IgG antibody (0.1–10 µg/mL) are shown in Fig. 6b2. This also indicated that color intensity was positively correlated with IgG concentration.

Then, the LoD for anti-SARS-CoV-2 IgM/IgG antibody was calculated after testing different concentrations (10–0.1 ng/mL) and the sigmoidal curves are shown in Fig. 6c1,c2. The LoD for IgM and IgG antibodies was 3 ng/mL and 7 ng/mL, respectively, calculated using the IUPAC standard method (LoD = mean value of the blank control + three times the standard deviation of the blank control)^55^. The results suggest that the developed novel patch had high sensitivity for SARS-CoV-2 IgM/IgG antibodies, which was comparable to or higher than that of commercial LFIA strips^56,57^.

## Discussion

Current detection methods for COVID-19, such as RT-PCR, require expensive laboratory-based facilities, trained laboratory technicians, and long turnaround time, which hinder mass screening and identification of infected individuals in developing countries with limited resources. Another method is to test SARS-CoV-2 specific IgM/IgG using LFIA as a complementary tool to expect recent or previous infection as well as confirm suspected COVID-19 cases with negative RT-PCR results. However, drawing blood sample causes pain to the examinees, and can lead to infection at the piercing site. To address these challenges, we developed a novel patch-type method for COVID-19 detection by integrating porous MNs and a new immunochromatographic assay. The proposed device facilitates minimally invasive and rapid detection of SARS-CoV-2-specific IgM/IgG antibodies by sampling ISF instead of blood. In addition, the COVID-19 detection patch can be self-applied without the assistance of medical personnel.

Currently, the existing work on MNs for ISF extraction and subsequent biosensing is mainly focused on hollow or swellable MNs. However, hollow MNs are commonly made of metal or non-biodegradable polymers that may harm human health, and swellable MNs require a laborious process for recovering extracted analytes^42^. In contrast, although porous MNs can resolve these issues, they pose challenges of complicated and time-consuming manufacturing processes. Therefore, we propose a novel and simple fabrication process for porous MNs composed of biodegradable PLA microspheres. The interconnected micropores were directly formed by the voids among the PLA microspheres that were subsequently heat-treated. Heat treatment caused the microspheres to melt and bond together, resulting in stable and robust porous structures. The advantage of the proposed fabrication method is that once PLA microspheres are formed, the spherical shape can be maintained stably in an ambient environment, moreover, they are amenable to scaled-up production. The optimal temperature for heat treatment was 180 °C, and the absorption volume of sample fluid (110 µL/array in 2 min) suggested sufficient absorption ability for biosensing.

In addition, taking advantage of ISF extraction in a vertical direction via capillary action, we designed and developed a paper-based immunoassay in a new structure incorporating vertical and lateral-flow structures to fit porous MN arrays and enable visualization of immunochromatographic results. Moreover, the length of the NC membrane was 2 cm to shorten the ISF flow distance, and the detection time was 3 min. In comparison, commercial LFIAs for IgM/IgG antibody tests require blood sampling, buffer dispensing, and a waiting time of 15 min. Therefore, our detection patch is compact, easy to use, and provides reliable results in a comparatively shorter time.

Besides, the sensitivity (LoD) of PMNIA was 3 ng/mL for IgM detection and 7 ng/mL for IgG detection, which is better than that of some of the current commercial AuNP-based LFIA kits detecting SARS-CoV-2-specific antibodies^58^. Therefore, the developed PMNIA provided a more accurate detection of SARS-CoV-2-specific IgM/IgG antibodies and reduced the false negative rate. Though SARS-CoV-2 specific antibodies in dermal ISF have not been extensively studied, previous studies on the proteomic analysis of body fluids have reported that compared with plasma and serum, ISF is highly homogeneous and identical in terms of protein diversity, including immunoglobulins^59,60^. Thus, we expect that anti-SARS-CoV-2 IgM/IgG antibody levels in ISF correlate well with those in the blood, and could be detected by the proposed PMNIA. In the future, clinical validation of the developed patch sensor will be conducted on infected and non-infected individuals who have not been vaccinated to investigate the specificity and sensitivity in comparison with RT-PCR as the gold standard for clinical diagnosis of COVID-19^20^. Besides, the comparison of detection performance of the new device with commercially available LFAs using blood sample and ISF sample will be carried out. It is envisioned that the minimally invasive, simple, and rapid detection patch shows great prospects for protein biomarker detection in ISF and diagnosing other infectious diseases, and can be widely employed in medical resource-limited settings.

## Conclusion

In this study, a novel patch-type COVID-19 diagnostic device, PMNIA, integrating porous MNs and an immunochromatographic biosensor was developed. A simple and novel fabrication method for biodegradable porous PLA MNs by emulsion and heat treatment was optimized to achieve the best absorbing ability and sufficient mechanical strength to pierce porcine and rat dorsal skin. The structure of the paper-based immunoassay was designed with vertical and lateral flow to realize continuous sampling from the MN array to the immunoassay biosensor and enable clear visualization of the detection results by the naked eye. An in vitro test was conducted using a model skin by applying the patch to agarose gel, and results were observed at the control line within 3 min. Moreover, the LoD for SARS-CoV-2-specific antibody detection indicated a high sensitivity compared to that of commercial LFIAs. We envision that people can detect IgM and IgG antibodies against SARS-CoV-2 painlessly and rapidly using this newly structured immunoassay. Furthermore, the compact size of the integrated diagnostic device is advantageous to both clinicians and patients. In addition, the proposed device has great potential for fast screening of various kinds of infectious diseases as an effective complementary method with other diagnostic tests.

## Materials and methods

### Reagents and materials

For the manufacture and assessment of the porous PLA MNs, PLA (Ingeo 4032D) was obtained from NatureWorks (Minneapolis, MN, USA). Dichloromethane (135-02446), trehalose dihydrate (202-18452), luminol (123-02583), hydrogen peroxide (081-04215) were purchased from Fujifilm Wako Pure Chemical (Osaka, Japan). Poly(vinyl alcohol) (363103), methylene blue (M9140), peroxidase from horseradish (SRE0082), 3,3′,5,5′-Tetramethylbenzidine (860336), glucose oxidase (G7141) and sodium carbonate (V800370) were obtained from Sigma-Aldrich (St. Louis, MO, USA). Polydimethylsiloxane (PDMS) pre-polymer and curing agent (Silpot 184) were purchased from Dow Chemical Company (Midland, MI, USA). Agarose gel (NE-AG01) was purchased from Nippon Genetics (Tokyo, Japan). Isoflurane was obtained from Pfizer (New York, NY, USA). Depilatory cream (LBS-s, Veet) was purchased from Reckitt Japan (Tokyo, Japan).

For the preparation of the paper-based immunoassay, the sample pad, conjugate pad (Standard 14), NC membrane (FF120 HP Plus), and absorption pad (CF4) were purchased from Whatman (Maidstone, UK). Hydrophobic membranes were obtained from LINTEC Co. (Tokyo, Japan). Gold colloids (AuH2, 40 nm) were purchased from Morinaga (Yokohama, Japan). The borate buffer was obtained from Fujifilm Wako Pure Chemical. SARS-CoV-2 spike protein RBD (C19SD-G241H), anti-SARS-CoV-2 spike Protein hIgM antibody (G19S1-M60H), anti-SARS-CoV-2 spike Protein hIgG antibody (G19S1-60H), mouse anti-human IgM monoclonal antibody (H38M-60M-1000), and mouse anti-human IgG monoclonal antibody (H38-60M-1000) were purchased from SignalChem (Richmond, Canada). Rabbit IgG antibody (I8140), bovine serum albumin (BSA, A7030), and polyoxyethylene (20) sorbitan monolaurate (Polysorbate 20 or Tween-20, P1379) were purchased from Sigma-Aldrich, and anti-rabbit IgG (H + L) antibody was obtained from Funakoshi (Tokyo, Japan). PBS was obtained from Nacalai Tesque (Kyoto, Japan).

### Preparation of PDMS MN female mold

The MN stainless-steel male mold was first designed and manufactured using wire electrical discharge machining. The MN array was designed in a 13 × 13 array with an MN tip distance of 1 mm, and each MN was designed in a pyramidal shape (1200 µm height) and a square base (620 µm width). A mixture of PDMS and curing agent (10:1, w/w) was cast onto the MN male mold followed by degassing and curing in an oven at 80 °C for 1 h. The PDMS female mold of the MNs was then detached from the stainless-steel mold and subsequently used to fabricate porous PLA MNs.

### Preparation of PLA microspheres

First, 6.7% (w/v) PLA in 15 mL dichloromethane was prepared as organic phase and blended into the water phase that contained 5% (w/v) polyvinyl alcohol (PVA) as a surfactant in deionized (DI) water. To obtain PLA microspheres, the mixture was agitated at 1000 rpm at 25 °C for 6 h until the dichloromethane was completely evaporated.

### Fabrication process of porous PLA MNs

The fabricated PLA microsphere solution was then poured into the prepared PDMS female mold. After vacuuming to fill the microspheres into the cavities, the entire mold was placed in a convection oven at 50 °C for 2 h to evaporate the solution and dry the PLA microsphere solution to form the MN structure. The MN array was then peeled off from the mold. Then, heat treatment at a temperature higher than the melting point of PLA (170 °C) was applied for 30 min to make microspheres melt and bond together. After drying and heat treatment at four different temperatures (170 °C, 180 °C, 190 °C and 200 °C).

### Measurement of the MN porosity

The porosity of the porous PLA MNs was measured using the water imbibition method and porous PLA films by comparing their masses before and after fluid extraction. First, the dry mass (*W*_*dry*_) was recorded after which, the prepared film was immersed in DI water; surface water was removed after the absorption was allowed till saturation. Subsequently, the mass was measured immediately and recorded as *W*_*wet*_. Porosity was calculated using the following equation:

1 $$Porosity(\boldsymbol{\%})=\frac{\frac{{{\varvec{W}}}_{{\varvec{w}}{\varvec{e}}{\varvec{t}}}-{{\varvec{W}}}_{{\varvec{d}}{\varvec{r}}{\varvec{y}}}}{{{\varvec{\rho}}}_{0}}}{\frac{{{\varvec{W}}}_{{\varvec{d}}{\varvec{r}}{\varvec{y}}}}{{{\varvec{\rho}}}_{{\varvec{p}}}}+\frac{{{\varvec{W}}}_{{\varvec{w}}{\varvec{e}}{\varvec{t}}}-{{\varvec{W}}}_{{\varvec{d}}{\varvec{r}}{\varvec{y}}}}{{{\varvec{\rho}}}_{0}}}$$

where ρ_p_ is the density of PLA (1.25 g/cm^3^) and ρ_0_ is the density of DI water (1.0 g/cm^3^).

### Mechanical tests

The failure force of the porous MNs after heat treatment at different temperatures was evaluated by performing axial compression tests using commercial force–displacement equipment (MX2-500N; Imada Inc., Toyohashi, Japan). A single porous MN was placed on a flat plate and compressed by a rigid metal surface at the rate of 2 mm/min. The compression force and displacement were continuously measured until the MN broke, and the buckling failure force was recorded as the force suddenly dropped upon needle failure.

According to the MN dimensions and experimental results of the failure forces, the Young’s modulus (E) of the porous PLA MNs was calculated based on the following equation^61,62^:

2 $$E=\frac{240{\pi }^{2}{L}^{2}P}{\langle 120\left\{{W}_{2}\left[{W}_{2}^{2}\left({W}_{2}-2{W}_{1}\right)+2{W}_{1}^{3}\right]-{W}_{1}^{4}\right\}+\pi \left\{\begin{array}{c}20\left[{W}_{2}\left({W}_{2}^{2}\left(-{W}_{2}+{W}_{1}\right)-{W}_{1}^{3}\right)+{W}_{1}^{4}\right]+\\ {\pi }^{2}\left[{W}_{2}\left({W}_{2}\left({W}_{2}\left({W}_{2}+{W}_{1}\right)+{W}_{2}^{2}\right)+{W}_{1}^{3}\right)+{W}_{1}^{4}\right]\end{array}\right\}\rangle }$$

where *L* represents the height of the pyramidal MN and *P* is the critical buckling load that also refers to the MN failure force^54^. *W*_*1*_ and *W*_*2*_ represent the width of the tip diameter and MN base, respectively. This equation has a boundary condition wherein, the MN base is fixed, and the MN tip can move freely. The fabricated porous MN array satisfied the fixed-free case.

### In vitro MN skin insertion

To evaluate the skin insertion capability of porous PLA MNs, porcine cadaver skin (K1270; Funakoshi, Tokyo, Japan) was used to mimic human skin because of its anatomical and physiological similarities^63^. Porous MNs were inserted into porcine skin for 1 min under finger pressure (~ 30 N) and then peeled off from the skin. Subsequently, the skin surface was stained with 1% (w/v) methylene blue for 15 min and then wiped with ethanol. The sites penetrated by MNs were identified using stereomicroscopy (Stereozoom S9D; Leica, Wetzlar, Germany). Based on the observation of stained spots, the PE was calculated using the following equation:

3 $$PE=\frac{Number \; of \; blue \; spots}{Number \; of \; microneedles (=169)}\times 100\%$$

### Preparations of attached papers and luminol solution for in vivo test

The circular blank paper was prepared from a filter paper (Grade 4; Whatman, Maidstone, UK) with a diameter of 5 mm. The circular glucose-sensitive paper (Grade 4) was prepared with glucose oxidase and horseradish peroxidase as enzymes and TMB as the chromogenic dye which have been commonly used as colorimetric assays for detection of glucose level^31,64^. Firstly, enzymatic agent was prepared by mixing 100 U/ml GO_X_ (145.2 U/mg) and 100 U/ml HRP (279 U/mg) in 1 mL PBS buffer with 250 mM trehalose as a stabilizer. Then, 4 µL of the prepared solution was pipetted onto the paper and dried at 25 °C. Next, the chromogenic agent was prepared by dissolving 15 mM TMB in 1 mL methanol and pipetted with 4 µL onto the same filter paper and dried at 25 °C. The preparation of the glucose-sensitive paper biosensor and the blank paper were completed and attached to the MN array with transparent adhesive tape covered as illustrated in Fig. S5a.

Luminol solution has been extensively employed to verify the presence of blood via chemiluminescent reaction^65^. The solution was prepared by dissolving 0.1 g luminol, 5 g anhydrous sodium carbonate, and 15 mL of 30% hydrogen peroxide solution in 100 mL of DI water and stored in the dark.

### In vivo MN skin insertion and recovery

Sprague–Dawley rats (CLEA, Tokyo, Japan) at the age of ten weeks were used to characterize skin recovery after MN treatment. First, the rat was placed and anesthetized in the induction chamber prefilled with 5% Isoflurane at a flow rate of 2.5 l/min using a rodent inhalant anesthetic apparatus (WP-SAA01; LMS, Tokyo, Japan). After induction of inhalation anesthesia, the rat was moved onto a heating plate to keep its body temperature. Then the rat was covered with a nose mask, and 2% Isoflurane at a flow rate of 2.5 l/min was applied from an in inhaler during hair removal and MN penetration test. The hair of the rat dorsal skin (2 cm × 2 cm) was shaved using an electric shaver and subsequently applied with 0.5 g depilatory cream for 1 min, resulting in the exposed rat skin after removal of the depilatory cream using wet tissues. In order to avoid physical damage to the rat when applied with a continuous force for MN insertion, the rat dorsal skin was fixed onto a 3D-printed PLA platform (Fig. 4d1). The porous MN array attached with prepared papers were placed on the dorsal skin and applied with a thumb force (~ 30 N) for 5 min and then peeled off for further observation. The prepared luminol solution was sprayed to the blank paper to confirm whether blood was present in the extracted fluid. The animal test was approved by the research ethics committee of Institute of Industrial Science, The University of Tokyo (the ethics approval number: 03–05 in 2021), and this study was performed in accordance with the ARRIVE guidelines.

### Preparation of AuNP conjugates

To prepare the SARS-CoV-2 spike protein RBD-conjugated AuNPs, 10 µg recombinant SARS-CoV-2 spike protein RBD was added to a mixture of 1 mL AuNP colloid (40 nm in diameter, OD = 12) and 0.1 mL borate buffer (0.1 M, pH 8.5) to facilitate the binding of proteins to the surface of AuNPs. After incubation 30 min at 25 °C, 100 µL BSA (10 mg/mL) was added to block the AuNP surface. After 15 min incubation at 25 °C, samples were centrifuged at 8600 g for 20 min at 4 °C. Then, the supernatant was discarded and 1 mL BSA (1 mg/mL) was added to resuspend the conjugated AuNPs; their surface was blocked again, and the unbound spike protein was removed. The centrifugation and resuspension steps were repeated twice and 1 mL PBS was used for the final resuspension. Rabbit IgG-conjugated AuNPs were prepared using the same procedure.

### Preparation of the paper-based immunoassay

The sample pad was prepared by cutting into a size of 1 cm × 1 cm and subsequently dipping into the pretreatment solution containing 0.5% (w/v) BSA and 0.05% (v/v) Tween-20 in PBS until fully wet. Then the sample pad was dried at 37 °C for 2 h in an oven.

The conjugate pad was cut into a size of 5 mm × 5 mm, pretreated with a buffer comprising 5% (w/v) sucrose, 1% (w/v) BSA, and 0.1% (v/v) Tween-20 and dried at 37 °C for 2 h. Here, sucrose was used to preserve the stability of protein after dehydration and improve quick re-solubilization upon wetting^66^. Then, the conjugate pad was successively coated with the prepared SAR-CoV-2 spike protein RBD-conjugated AuNPs and rabbit IgG-conjugated AuNPs and then dried at 37 °C.

The capture anti-human IgM antibody, anti-human IgG antibody, and anti-rabbit IgG antibody, were immobilized on the NC membrane (4 mm × 2 cm) using a polyester swab with a sharp tip (200-CD055; Sanwa Direct, Okayama, Japan) as the IgM test, IgG test, and control lines, respectively. The IgM line was printed 7 mm from one end of the NC membrane and the distance between the two lines was 4 mm. Afterwards, the NC membrane was blocked with 2% (w/v) BSA in PBS followed by washing with 0.05% (v/v) Tween-20 in PBS, and then dried at 37 °C for 30 min. The adsorption pad, without any treatment, was attached, with 1–2 mm overlapping with the NC membrane.

### Quantitative analysis of the paper-based immunoassay

After the colorimetric signal in the test and control lines stabilized, the detection patches were placed in a box, and pictures were taken using a camera (D5500; Nikon, Tokyo, Japan). The setup conditions, including the camera parameters, light source, and distance between the detection patch and camera, were fixed and kept constant during imaging. Next, the image was imported into ImageJ (National Institute of Health, Bethesda, MD, USA), and the green channel was selected because it provides the highest sensitivity for the analysis of red labels, such as AuNPs^66^. A straight line was drawn along the NC membrane. The width of the straight line was adjusted to cover most of the strip to increase reproducibility. Then, the peak intensity of test lines (IgM or IgG) after testing different concentrations was measured by subtracting the peak intensity value from the background intensity value. Subsequently, the data were imported into the Origin software (2021b; OriginLab, Northampton, MA, USA), and the best data fitting was obtained using a four-parameter logistic curve for statistical analysis including LoD determination for IgM/IgG. All methods including animal experiments were carried out in accordance with relevant guidelines and regulations (the ARRIVE guidelines 2.0), and as mentioned before, all animal tests were approved by the research ethics committee of Institute of Industrial Science, The University of Tokyo, Japan.

## Supplementary Information

Supplementary Figures.

## Data Availability

All data generated or analyzed during this study are included in this published article (and its supplementary information files).
