# Supplementary material for: Anti-SARS-CoV-2 IgM/IgG antibodies detection using a patch sensor containing porous microneedles and a paper-based immunoassay
Source: Sci Rep. 2022 Jul 1;12:10693. doi: 10.1038/s41598-022-14725-6 (PMC9249772; doi:10.1038/s41598-022-14725-6)
Supplement: Supplementary file 1 — Supplementary Figures. [file 41598_2022_14725_MOESM1_ESM.docx]

Supplementary information for the paper:

Anti-SARS-CoV-2 IgM/IgG antibodies detection using a patch sensor containing porous microneedles and a paper-based immunoassay

Leilei Bao, Jongho Park, Boyu Qin, and Beomjoon Kim^*^

Institute of Industrial Science, The University of Tokyo, 4-6-1 Komaba, Meguro-ku, Tokyo 153-8505, Japan

*Corresponding author: Beomjoon Kim (bjoonkim@iis.u-tokyo.ac.jp)

**Supplementary Figures:**


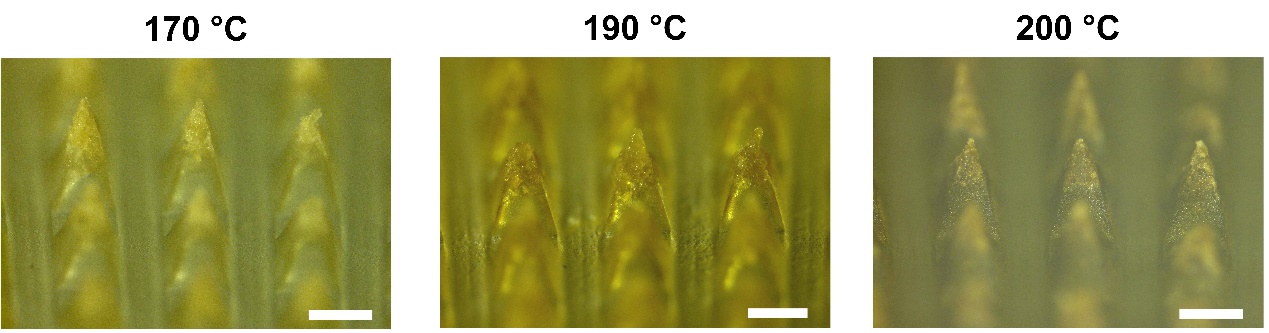


Fig. S1 Microscopic images of porous PLA MNs shapes after heat treatment at different temperatures. Scale bar, 500 μm.


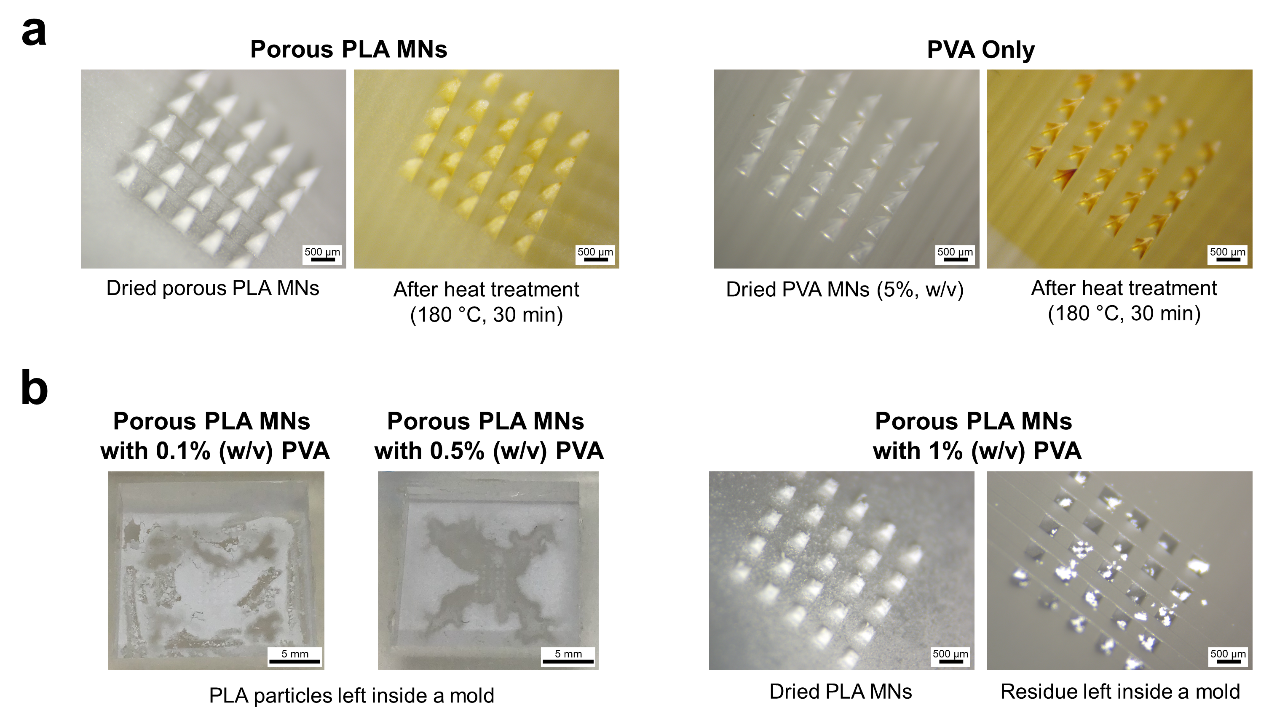
Fig. S2 Microscopic images of porous PLA MNs fabricated from PVA solutions with different concentrations. **a** Comparison of porous PLA MNs and PVA MNs only. Color changes were observed in both cases **b** Fabrication of PLA MNs using PVA solutions with three different concentrations, 0.1, 0.5, and 1%. Porous PLA MNs were not formed and maintained for three concentrations.


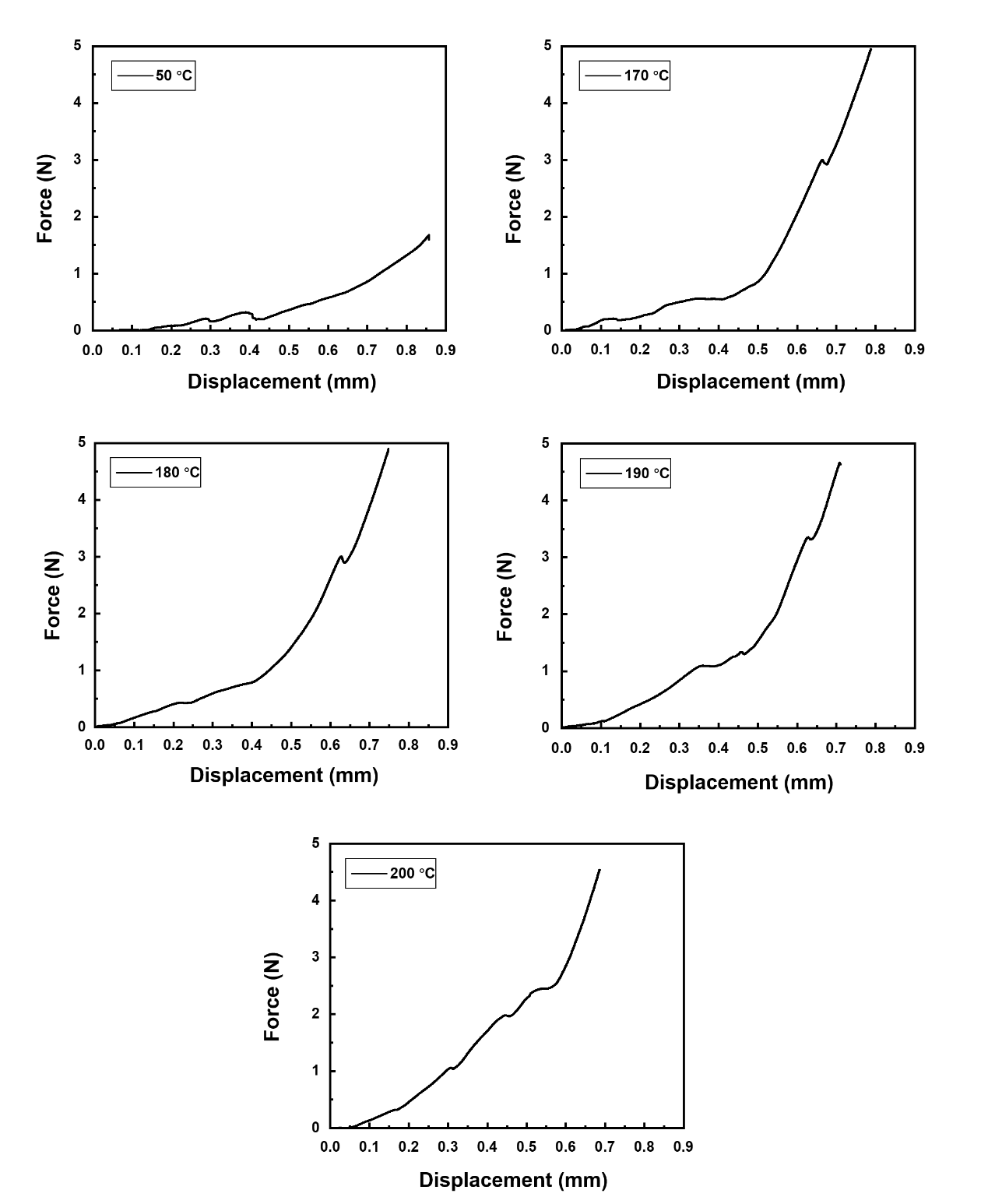


Fig. S3 Representative force-displacement curves of uniaxial compression tests for porous PLA MNs after heat treatment at different temparatures.


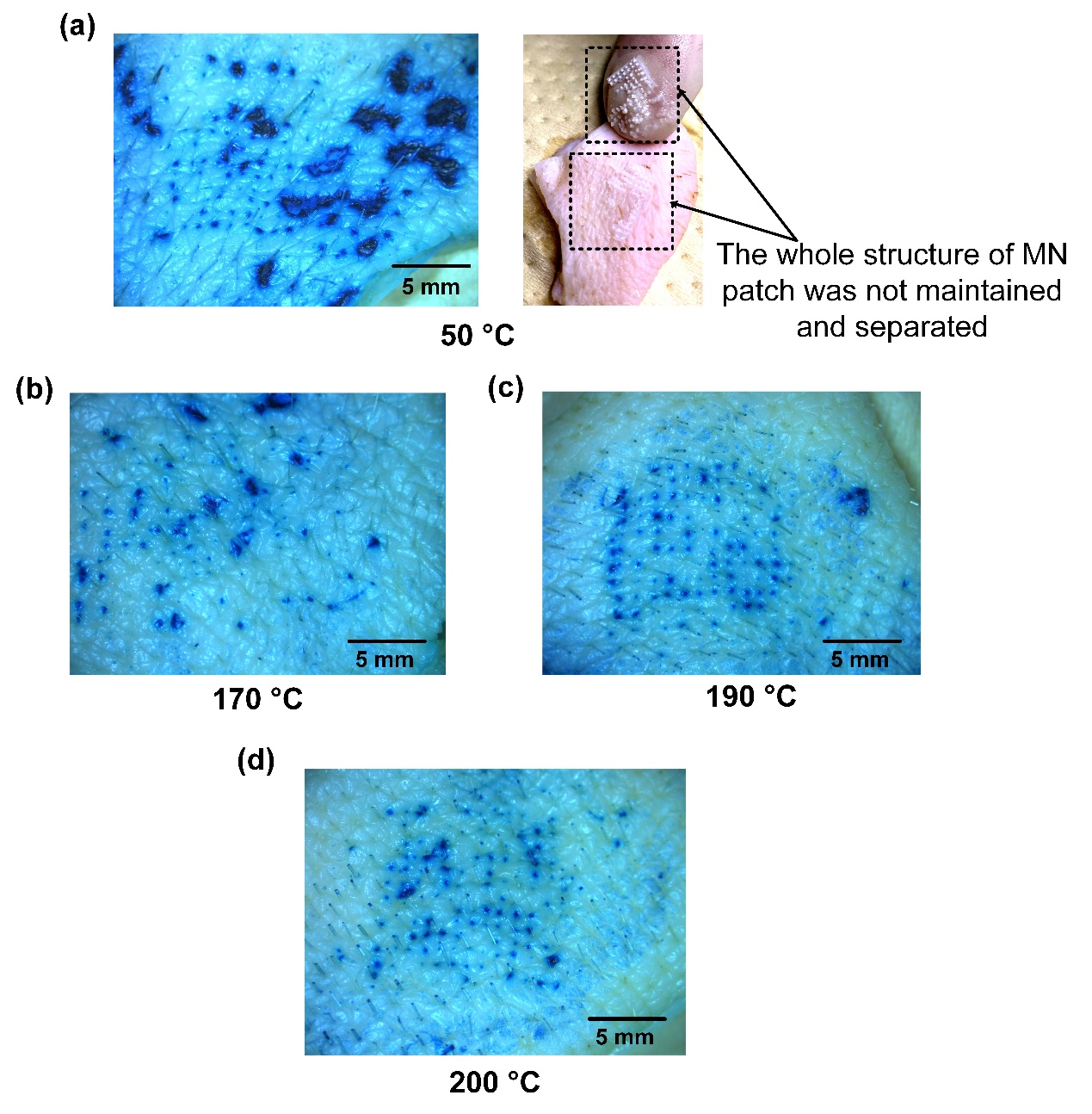


Fig. S4 Representative images of skin penetration tests using PLA porous MNs after heat treatment at different temperatures.


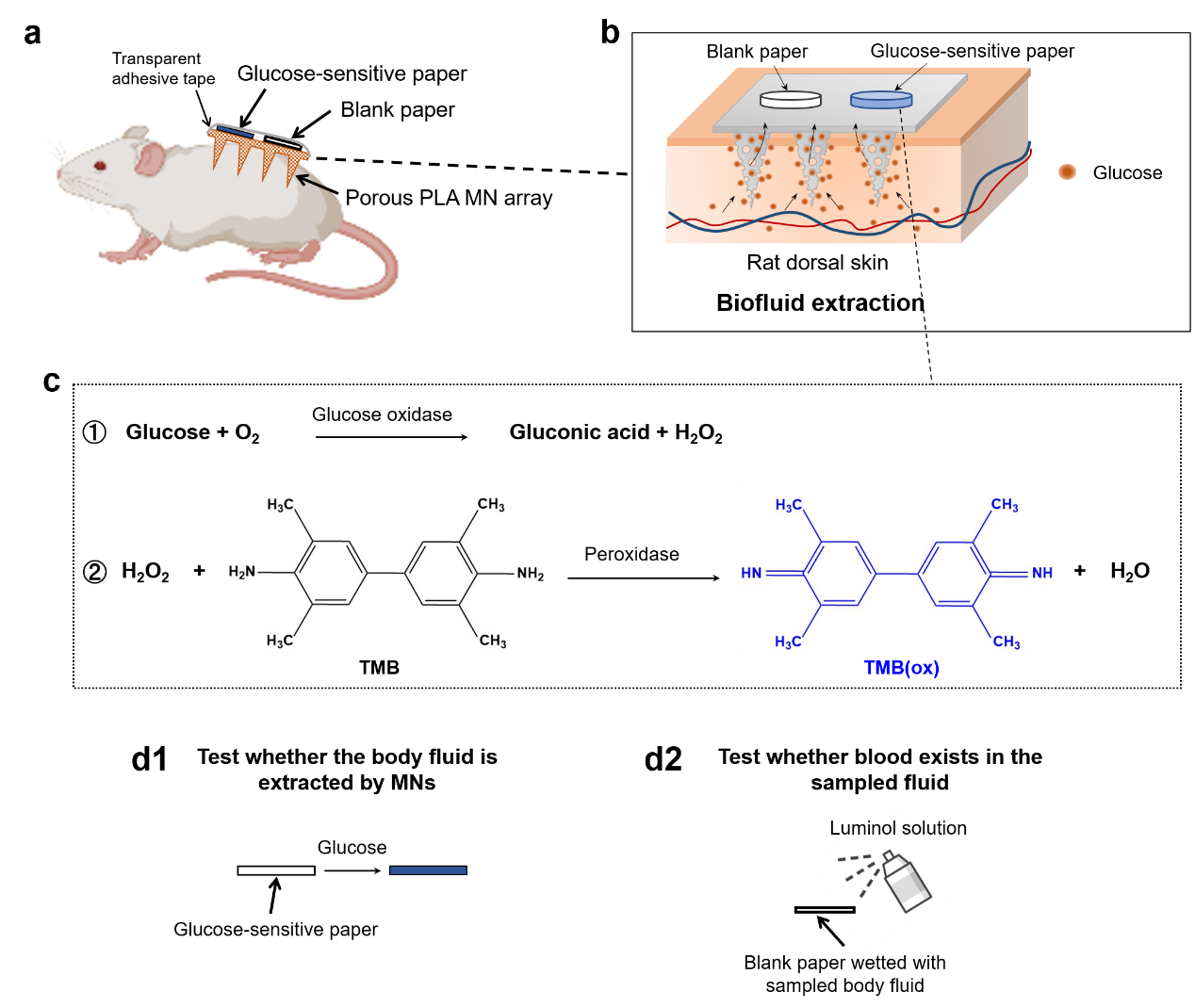


Fig. S5 Schematic of evaluation of ISF extraction using porous PLA MNs via *in vivo* test. **a** Insertion of porous MNs with the prepared glucose-sensitive paper and blank paper attached into the rat dorsal skin. **b** The blank paper gets wetted and the glucose-sensitive paper appears blue color due to the colorimetric reaction if the ISF containing glucose is extracted and transported via MNs to the attached papers. **c** The procedure of colorimetric assay reaction at the sensor layer where the enzymes (glucose oxidase and horseradish peroxidase) and chromogenic dye (TMB) react with the extracted glucose. **d** Observation of ISF extraction result. The glucose-sensitive paper is applied to assess the fluid extraction performance (d1). The blank paper is sprayed with luminol solution to test whether blood is contained in the sampled body fluid (d2).


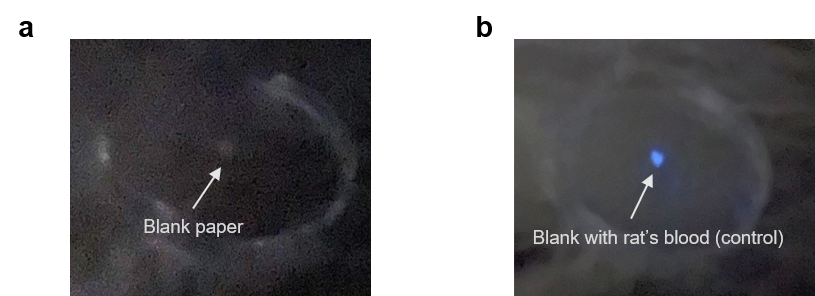


Fig S6 Spray with luminol solution. **a** Absence of chemiluminescence reaction of the blank paper. **b** Blue light emitted from the blank paper dipped with a drop of rat blood as control.
